# Bioinformatic pipelines in Python with Leaf

**DOI:** 10.1186/1471-2105-14-201

**Published:** 2013-06-21

**Authors:** Francesco Napolitano, Renato Mariani-Costantini, Roberto Tagliaferri

**Affiliations:** 1 Department of Computer Science (DI),University of Salerno, Fisciano (SA) 84084, Italy; 2 Department of Medicine, Dentistry and Biotechnology “G. d’Annunzio” University, Chieti-Pescara, Italy; 3 Unit of General Pathology, Aging Research Center (CeSI) “G. d’Annunzio” University Foundation, Via Luigi Polacchi 15/17, Chieti 66100, Italy

**Keywords:** Data analysis, Bioinformatic pipelines, Python

## Abstract

**Background:**

An incremental, loosely planned development approach is often used in bioinformatic studies when dealing with custom data analysis in a rapidly changing environment. Unfortunately, the lack of a rigorous software structuring can undermine the maintainability, communicability and replicability of the process. To ameliorate this problem we propose the Leaf system, the aim of which is to seamlessly introduce the pipeline formality on top of a dynamical development process with minimum overhead for the programmer, thus providing a simple layer of software structuring.

**Results:**

Leaf includes a formal language for the definition of pipelines with code that can be transparently inserted into the user’s Python code. Its syntax is designed to visually highlight dependencies in the pipeline structure it defines. While encouraging the developer to think in terms of bioinformatic pipelines, Leaf supports a number of automated features including data and session persistence, consistency checks between steps of the analysis, processing optimization and publication of the analytic protocol in the form of a hypertext.

**Conclusions:**

Leaf offers a powerful balance between plan-driven and change-driven development environments in the design, management and communication of bioinformatic pipelines. Its unique features make it a valuable alternative to other related tools.

## Background

**Figure 1:**
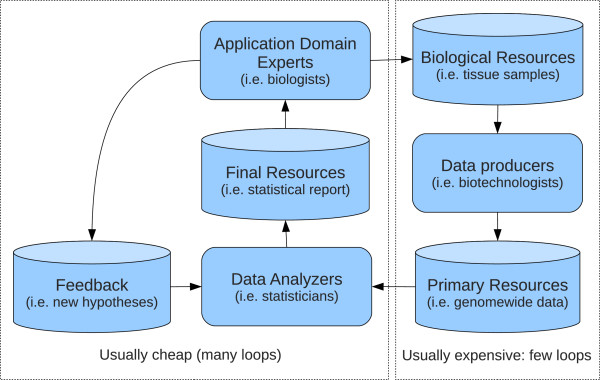
**Example of bioinformatic collaboration.** Bioinformatic research collaboration scheme. This is a representation of a typical process in a bioinformatic study where different research units are involved. The left part of the scheme represents a loop with iterations that are usually cheaper (no consumables or expensive technologies involved) and more prone to be iterated over time in order to refine the analysis based on partial results. This loop represents a major challenge in terms of code maintainability.

Systemic Bioinformatic analysis requires heterogeneously composed research groups, including data producers, data miners and application domain experts (such as biologists). Data producers use dedicated technology on biological specimen to extract data; data miners analyze data and try to highlight relevant information; biologists examine the filtered data, which thy then validate through targeted experiments and use to support their hypothesis or to formulate new ones (See Figure 1). Custom bioinformatic analysis requires programmers to implement new methods and/or put together existing ones in order to build new data analysis frameworks (data flows [1], commonly known as bioinformatic pipelines). In such cases, high level scripting languages are used (such as Python, Perl, R, Matlab) to quickly implement and test new methodologies and present results to other research groups, while statically typed languages (like C, C++, Java) are generally preferred when computational performance is crucial [2-4].

Indeed the priority of custom data analysis software is above all about results, while features like code maintainability, documentation and portability are often considered secondary. In our experience, a precise design of the analysis process is usually impossible to achieve in advance, since the feedback produced by preliminary results may drive the study in unpredictable directions. In fact, the main shortcoming of plan-based development paradigms is in their lack of responsiveness to change. Software Engineering deals with such issue by means of dedicated development models (Extreme Programming, Prototype-based Programming [5], Agile development [6]) that try to relax formal constraints in order to more easily adapt to dynamic conditions [6].

However, if taken to an extreme, prototype-based approaches tend to undermine the integrity of the system’s architecture [7], accumulating patches as more requests are fulfilled. The resulting analysis is often hard to reproduce, which is also due to difficulties with establishing its execution provenance [8-10]. Such challenges have been recently evinced in [11], where urgency for open source code in scientific development was emphasized as a consequence of the difficulty of reproducing bioinformatic results and accounting for all the technical aspects of a bioinformatic analysis. In addition, we note that loosely structured and poorly documented processes can hardly be reproduced even when source code is available.

**Figure 2:**
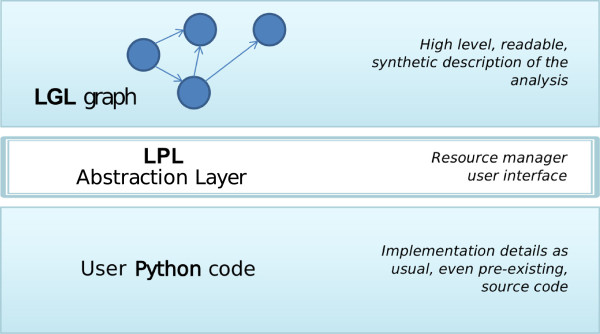
**Leaf architecture.** A Leaf project is carried out by three different layers. On the top layer, a high level definition of a pipeline is coded through the Leaf Graph Language (LGL). The Pyleaf library is able to interpret an LGL graph as a computational pipeline and bind the nodes of the pipeline to the user’s Python code.

**Figure 3:**
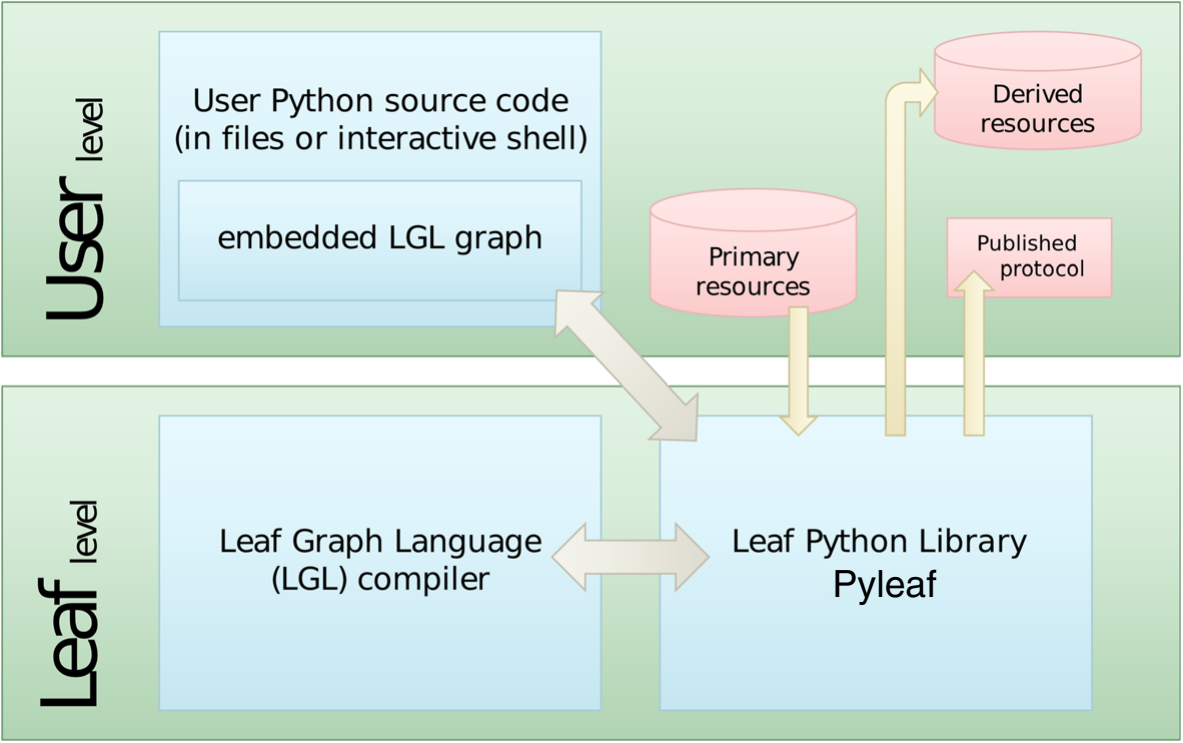
**User interaction.** The interaction between the user’s Python code and the Leaf system happens through the definition of an LGL graph (the high level pipeline) and the use of Pyleaf. The user creates a new Leaf project by providing Pyleaf with the pipeline definition, the Python code implementing each node of the pipeline and the primary resources to work on. The project is then used to request the production of resources.

**Figure 4:**
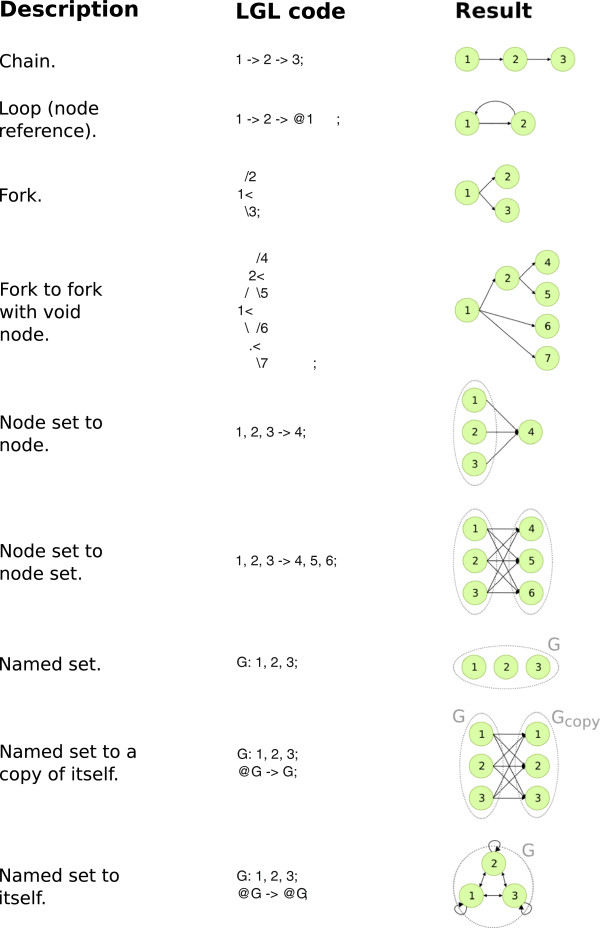
**LGL syntax examples.** This figure shows some examples of graph structures defined through the Leaf Graph Language (LGL). Tree structures of any depth and degree can be encoded using the fork arrow (<) and void node (.) mechanisms. Note that through LGL it is possible to define graphs that are not DAGs (see main text), though they are not allowed by Pyleaf.

In order to take into account the need to reduce planning and at the same time maintain high level structuring in bioinformatic analyses, we developed the *Leaf *[12,13] system (Figures 2 and 3). Its purpose is to allow for the transparent association of regular code written in a high-level programming languages with a pipeline structure. To this aim, Leaf implements a graph design language (namely LGL, Leaf Graph Language, see Figure 4 for a brief overview) that allows the programmer to define the high level pipeline describing his analysis directly within his source code in other languages. Specifically, we developed LGL support for the Python language, implemented as the Pyleaf library. Python was chosen as a high level, dynamically typed, general purpose, interpreted language, that offers interoperability with other languages (like R) and clean, readable code [2]. Moreover, a growing community of Bioinformaticians has shown interest in the Python language, also through the development of tools like those collected in the Biopython project [14]. The LGL language provides an extremely minimalist approach, enforcing almost no conventions as to how the pipeline nodes must be implemented.

The formal definition of a pipeline is used by Leaf to provide a number of automated features described in the next sections. As a final output, Leaf is able to generate a hypertext document describing both the high level design and all the details of the analysis (the *bioinformatic protocol*), including provenance information.

### Previous work

The development of data flow management systems has recently become a very active area in bioinformatics [15]. Though an extensive review of the existing tools is out of the scope of this paper, a simple overview will be useful in order to identify the strengths and weaknesses of Leaf with respect to related software. In order to correctly place Leaf in the landscape of other tools, we divide them into three categories, based on the thickness of the abstraction layer between the pipeline definition and its implementation. Of course the three categories are not perfectly disjoint, as some tools may cross their boundaries.

In general, the most high-level tools try to encapsulate implementation details as much as possible in order to facilitate interactions with the top layer of the analysis. The user is presented with a Graphical User Interface and builds a bioinformatic pipeline with point-and-click applications. Examples of graphical approaches include Galaxy [16], Taverna [17], Pegasys [18], Conveyor [19], Kepler [20]. Such tools often include support for extending their functionality through either dedicated or existing scripting languages, allowing for additional flexibility. However, their main focus and strength is about simplifying the management of the data flow structure, while lower level changes are not as immediate. Most of these tools are also suitable for researchers without programming skills.

A thinner abstraction layer is provided by a number of pipeline design tools based on formal languages, such as Anduril [21] and Biopipe [22]. The purpose of these tools is to provide robust general frameworks for data analysis that can recognize and integrate heterogeneous data structures. Unlike the higher-level tools, these approaches usually require the user to make the effort of formally describing data formats and processing semantics, after learning the adopted conventions. Their strength is in their ability to ensure the robustness of the processes while at the same time granting access to low level details.

The thinnest abstraction layers allow the user to easily bypass the pipeline manager itself, if needed. Leaf sits in this category, together with other tools like Ruffus [23] and Bpipe [24]. These systems are specifically designed for users with programming skills and their aim is neither to broaden pipeline management accessibility to a wider audience nor to guarantee a particular robustness of the process through the rigorous specification of data and exchange formats. On the contrary, their aim is to provide an easy data flow management support within existing development environments in order to simplify the coding process and encourage direct modifications of low level details. They are usually the most light-weight tools among data flow managers and are implemented as libraries or ad-hoc scripting languages. In the following we justify the development of Leaf by detailing the main differences between it and the other tools in the same category just mentioned: Bpipe and Ruffus.

**Figure 5:**
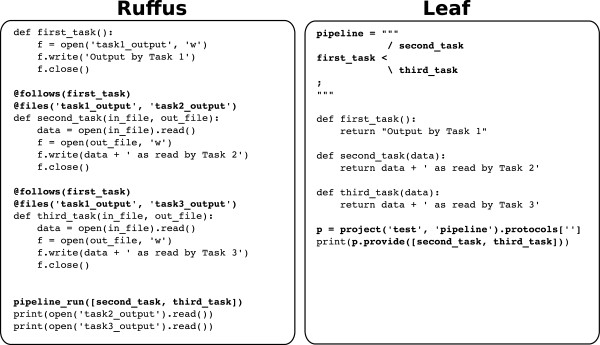
**Comparison between Ruffus and Leaf code.** Code for pipeline definition and use is highlighted in bold. Both samples implement the same simple pipeline made of three nodes (namely first_task, second_task and third_task), the first passes a text string to the other two, which in turn append some additional text to it. In Ruffus (left) the pipeline structure is defined through the “@follows” decorator, which must be attached to each function definition in order to specify its ascendants. In Leaf (right) the pipeline structure is visually defined as standalone LGL code (first lines in the example). Ruffus keeps track of produced resources by checking files specified through the “@files” decorator, which is the main tool for exchanging data between nodes. Leaf uses common function parameters while seamlessly caching their content on the disk to track the processing status. Leaf also caches the source code that produces each resource, and is thus able to detect changes in the code and invalidate all the resources affected by the change. The Ruffus file-based mechanism is also supported by Leaf through the “F” flag in LGL with the only requirement being that the function producing the files as their resources must return the corresponding file names (see main text).

The major shortcoming of Ruffus has been highlighted in [24] as their own motivation: the syntactic mechanism of Python “decoration”, which is the basis of Ruffus implementation, spreads the design of the pipeline structure throughout the code. Tasks like reading or modifying the data flow structure can be quite involved, since no general overview of the pipeline is actually present in the code. Conversely, the LGL formal language developed for Leaf is intended to be intuitively readable, having the unique feature that its code visually represents the pipeline it encodes (Figure 4). Most importantly, the Leaf system keeps the definition of the pipeline in one place and completely separated from the Python code implementing the single nodes (though still in the same Python source file). The LGL is a dedicated language with its own syntax, thus requiring some additional effort that is not required for Ruffus. However, the overall learning curves of the two systems on the whole are comparable. It should also be pointed out that an advanced LGL coding style, while producing visually rich code, also makes it harder to maintain. This is why, in addition to complex syntactic constructs, we also provided simple shortcuts, which allow the programmer to choose his preferred level of balance between easy to read and easy to write code. Figure 5 shows a comparison between two Leaf and Ruffus code fragments and describes some additional Leaf features that are not currently supported by Ruffus.

Like Leaf, Bpipe includes a dedicated language to define pipeline structures. However, Bpipe is primarily intended to be a pipeline-oriented replacement of shell scripts, built to run a pipeline of system commands that exchange data through files on the disk. This approach is the most straightforward, for example, in any environment where nodes of the pipeline are standalone programs. On the contrary, Leaf is meant to provide pipeline management support for general purpose scripting languages, such as Python. Nodes are implemented as functions that can exchange structured variables of arbitrary complexity in primary memory (the use of files is optional). With Leaf (and Ruffus), the definition of such functions can be provided together with the pipeline structure in the same source file.

## Concepts

In this section we introduce the concepts that formalizes the idea of bioinformatic pipeline implemented in Leaf. A Leaf pipeline is designed incrementally throughout the development phase of a bioinformatic analysis. Once the analysis is completed, a final protocol can be generated that documents the analysis process.

### Resources and processors

In our view, there are two kinds of actors in a data analysis process: Resources and processors. Resources are any type of data, including raw data, processed data, images, files etc. Here, by “raw data” we mean data at any stage that does not represent a final result of the analysis. Processors are computer routines that can modify existing resources or create new ones.

We subsequently distinguish between *primary* and *derived* resources. Primary resources are the initial input to the process and should be regarded as the ground truth of the analysis. Derived resources are obtained exclusively as automatic elaborations of primary resources. Exceptions to this constraint, like the introduction of manual interventions or implicit dependence on resources that are not part of the pipeline, could cause any automatic consistency check to fail. Derived resources can be further divided into *raw* resources (representing data at an intermediate processing stage) and *final* resources (representing the results of the analysis).

### Bioinformatic protocols as annotated DAGs

A graph [25] is an ordered couple (*V*,*E*), where *V* is a set of nodes and *E* ∈ *V* × *V* is a set of edges between nodes. Let us consider processors as nodes in a graph and resources as edges, such that, for example, a graph (*V* = {*x*,*y*,*z*}, *E* = {(*x*,*y*),(*y*,*z*)}) represents a data flow where the processor *x* provides (imports or generates) a primary resource passed through the edge (*x*,*y*) to the node *y*. The node *y* produces the raw derived resource passed through the node (*y*,*z*) to the node *z*. The node *z* produces a final derived resource. In our context a graph describing a data flow must be directed and acyclic (DAG).

We define a bioinformatic protocol as an annotated DAG. Here, annotations are all the details that are necessary to understand and execute the procedure described by the graph, including source code and documentation of the processors and the produced resources. Leaf protocols also include statistics detailing the time and space consumed by the execution of each node. Finally, since the actual implementation of Leaf requires each node to produce only one resource (even though it can be a structured object), the association of resources with edges is equivalent to the association of resources with nodes.

## Implementation

**Figure 6:**
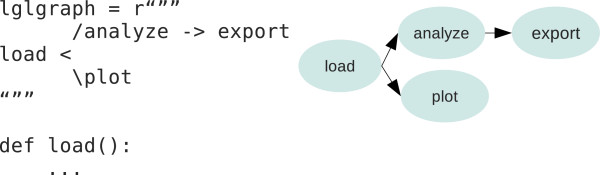
**Source code embedded pipeline definition.** Any programming language supporting multi-line text can include the definition of a pipeline as LGL code. Left: a Python example including an LGL graph definition whose nodes are implemented in the same source file. Right: a graphic representation of the corresponding pipeline.

**Figure 7:**
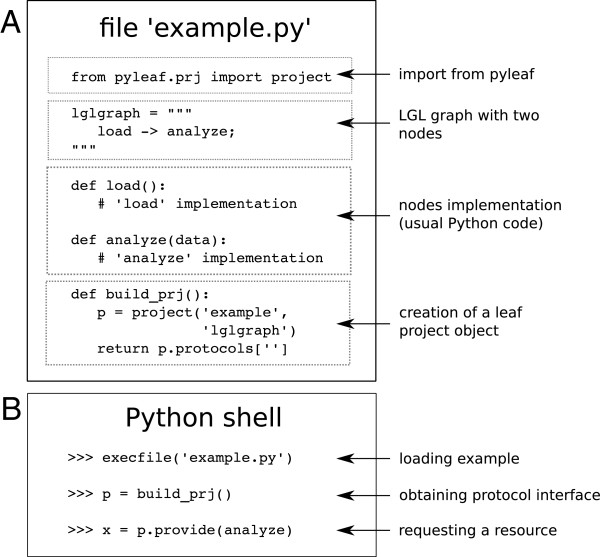
**Coexistence of leaf and python.** (**A**) Python source code including a pipeline definition as an LGL graph (Section “Concepts” in the source code), the implementation of the corresponding nodes and a function creating and returning a Leaf project object accordingly. (**B**) Example of a Python interactive session where the user loads the previous Python code, creates the Leaf project and request the production of a resource. The user can directly call pipeline nodes as regular Python functions by passing the input parameters manually. Leaf can call them automatically for the production of a resource as necessary. The thinness of the Pyleaf library abstraction layer allows for quick prototyping and experimentation.

Leaf is a software tool that supports the generation and use of bioinformatic pipelines as defined in the previous section. The Leaf system is composed of two subsystems (see Figure 2): the Leaf Graph Language (LGL) and the Pyleaf Python library, which are described in the following subsections. The user provides the description of a graph in LGL language together with the Python source code implementing the nodes. Then he has access to Leaf features through the Pyleaf interface (see Figure 3). Pyleaf transparently runs an external compiler that translates the LGL code into a simpler description of a graph structure. It then uses this structure to understand dependencies between nodes in the pipeline and to run the necessary nodes according to the user’s requests. In general, LGL source code is meant to be embedded into source code of other languages (see Figure 6) and exploited through ad hoc libraries. Pyleaf is the library that implements the Leaf system for the Python language (see Figure 7).

The LGL compiler (lglc) was built using the *Flex *[26] lexical analyzer and the *Bison *[27] parser generator. The compiler currently supports basic error handling, pointing out the line of code where a syntax error is detected. This proves very useful when editing complex graphs. On the other hand, errors detected in the node implementations are handled by the Python interpreter as usual: Leaf does not interfere with this mechanism other than adding its methods to the call stack.

Graph visualizations are produced using *Graphviz* tools [28]. More details can be found on the home page of Leaf [12] and on its public source code repository [13].

### The leaf graph language

The Leaf Graph Language (LGL) is a formal language with the unique feature of having a graphical appearance even though it is written in regular text. The purpose of LGL is to encode general graph structures (including graphs containing cycles, which are not supported by Pyleaf). An LGL graph definition can be directly embedded in the code of other programming languages to serve as a high level pipeline description. While a formal description of the language’s grammar is beyond the scope of this paper, in this subsection we present a few examples as well as the main syntax rules in order to illustrate its basic philosophy.

The fundamental objects in LGL are items, item sets, and arrows connecting items or item sets. Each item may represent a graph node or an entire graph by itself, while an arrow may represent a single edge or a set of edges. For example, the statement:

is an LGL instruction that creates a graph with three nodes, *A*,*B* and *C*, and connects node A (single item) to the set of nodes composed of B and C (item set). LGL automatically translates an arrow into a set of graph edges according to the type and number of items. In the previous example the arrow is translated into the set of edges {(*A*,*B*),(*A*,*C*)}. The left arrow is also allowed:

which defines the set of edges {(*B*,*A*),(*C*,*A*)}. Formally, this happens based on the fact that the comma operator takes precedence over the arrow operator.

An item can represent a complex object when using named graphs. A named graph is a graph preceded by a text label and a colon, like in the following statement:

After the definition of a named graph, its label (G1 in this example) can be used wherever an item can be used: the compiler will replace it with a copy of the graph it represents. In particular, the statement D -> G1; creates a new graph *G*_2_(*V*_2_,*E*_2_), where *V*_2_ = {*A*,*B*,*C*,*D*} and *E*_2_ = {(*A*,*B*),(*A*,*C*),(*D*,*A*)}. This happens because in this case the arrow operator connects the new node D to all the root nodes (nodes with no incoming edges) of G1. Analogously, the statement G1 -> D; creates a new graph *G*_3_(*V*_3_,*E*_3_), where *V*_3_ = *V*_2_ = {*A*,*B*,*C*,*D*} and *E*_3_ = {(*A*,*B*),(*A*,*C*),(*B*,*D*),(*C*,*D*)}. In this case the arrow operator connects all G1 leaves to D. Note that the sequence of instructions:

creates three graph objects: the named graph G1 and two unnamed graphs (corresponding to *G*_2_ and *G*_3_ which are previously defined). This is because as the LGL compiler encounters a previously defined label it creates a new copy of the corresponding object. If the intention is instead to create a unique graph by incrementally adding nodes and edges, it must be explicitly stated through the mechanism of object reference. From the syntactic point of view this simply amounts to prefixing each referenced object with the @ symbol. When a reference object is encountered, the previously defined object with the same name is used instead of a new copy. For example, the following code:

creates a single graph *G*_4_(*V*_4_,*E*_4_), where *V*_4_ = {*A*,*B*,*C*,*D*} and *E*_3_ = {(*A*,*B*),(*A*,*C*),(*D*,*A*),(*B*,*D*),(*C*,*D*)}. Note that the @ symbol is used only with items defined in previous statements.

The mechanisms described above are sufficient to describe any graph in LGL. However, another syntax construct, the fork arrow, is introduced to improve readability. The fork arrow allows the user to define a tree structure that is visually evident. The G1 graph, indeed, can be equivalently defined in LGL as follows:

The fork operator is composed of the *less-than* character (<), visually representing a binary split, and the *slash* (/) and *backslash* (\) characters signaling the beginning of a left child and a right child. LGL syntax includes mechanisms to nest fork structures at arbitrary levels and with any number of children per level. See Figure 4 for additional examples. In addition, arrows can be mixed with forks. Let us consider the following example:

Any fork can indeed be redefined as an arrow. Finally, special flags can be associated with nodes by enclosing them in square brackets, as in the following example:

This instruction creates the graph G1 and associates the flag *F* to its node B. The *F* flag tells Pyleaf (described in the next subsection) to consider the output of the associated processor as a file name and to support dedicated features.

### Pyleaf

Pyleaf is a Python library that is able to bind the node names of an LGL graph to Python functions in order to interpret it as an analysis pipeline. As previously mentioned, the semantics of the graph see nodes as processors and edges as input/output connections between them. Root nodes are meant to be associated with primary resources, terminal nodes with final resources, and other nodes with raw resources. With Pyleaf the user can request the production of a resource by identifying it directly with the name of the processor producing it.

For Pyleaf to work two objects are needed: the pipeline structure in the form of a multi-line Python string containing a graph in LGL code; and the name of the Python source code file where the functions implementing the pipeline nodes are defined. Indeed, the binding between the nodes and the corresponding Python functions is performed by searching for LGL nodes and Python functions having the same name. With this information Pyleaf can build a leaf.prj.project Python object, which is the main interface to all of Leaf’s features (see Table 1 for a summary of the main implemented methods). Let us consider the following piece of Python code as an example:

**Table 1:** Protocol methods summary

**Method**	**Description**
clear	Clears a resource from RAM.
clearall	Clears all resources from RAM.
dumpOff	Switches dumping OFF.
dumpOn	Switches dumping ON.
export	Exports the graph to a pdf file, including docstrings.
getinputs	Collects all input resources that are input to the given node and returns a copy of them in a list.
list	Lists the state (unavailable / dumped / to be built) of all resources.
provide	Provides a resource. The resource is returned if available, loaded from disk if dumped, built on the fly otherwise.
publish	Exports the analysis as an HTML bioinformatic protocol.
rebuild	Clears a resource, then provides it.
run	Provides all leaf (final) resources.
trust	Assigns a resource to a node without invalidating dependent resources.
undump	Clears a dumped resource from the disk.
undumpall	Clears all dumped resources from the disk.
untrust	Clears a resource and all its dependent.

This methods are designed to be used through a Python shell to perform pipeline operations. *Dumping* is the automatic management of produced resources in permanent memory. A resource is said to be *available* if present in primary memory, *dumped* if previuosly stored on the disk.

In order to create the object pr, Pyleaf passes the lglGraph object to the LGL compiler, reads the resulting graph structure and searches the ex1.py file for the Python functions loadData, visualize, analyze and exportResults. The pr object is a high-level interface that primarily deals with analyzing the user’s code in order to create one or more protocol objects (a Leaf feature that is currently under development will allow the user to create and manage variants of a protocol). In order to easily access the protocol object, the following code is used:

The interaction between functions bound to pipeline nodes happens as follows. Each function has only one output resource, though it can be an arbitrarily structured object, as usual in programming languages. If a node in the pipeline has more than one outgoing edge, it will provide the same resource along each edge. On the other hand, a node having *N* incoming edges in the pipeline must be bound to a function having *N* input parameters and is called accordingly. This is an alternative to the approach where nodes with multiple inputs correspond to the same function called multiple times with different inputs. Leaf supports both semantic styles: to use the latter, multiple copies of the same node can be added to the pipeline (this is permitted by LGL syntax), where each one is connected with different input nodes. However, we prefer the former approach, that uses multiple inputs and a single output per function, as it tends to align more naturally with common programming practices.

When the user requests a resource, Pyleaf is able to identify the part of the pipeline that needs to be executed in order to build the resource. A sequence of function calls is thus performed according to the pipeline structure, where the output of each node forwarded to all of its descendants, according to the rules explained above. In order to optimize pipeline execution, Pyleaf supports parallel processing of independent nodes and mechanisms for “lazy processing”, which means it doesn’t execute processors that are not required to satisfy a user request. When referring to “unnecessary nodes” we mean nodes that are not in the path between a primary resource and the requested resource, as well as nodes that are in said path but whose output has been previously computed (and whose source code has not changed since then). This is possible because Pyleaf automatically stores all derived resources in primary and permanent memory as soon as they are produced. As an example, let us suppose that a user requests the production of the resource that is produced by the processor analyze from the previous example. This is done through the Python shell using the Pyleaf provide method:

Pyleaf will refer to the protocol’s graph and run the loadData function accordingly (with no argument), pass its output to the analyze function and return the result in the variable x. Both outputs will be transparently stored in primary and permanent memory (even if the output was not assigned to a variable). If the user later requests, for example, the resource produced by exportResults, Pyleaf will load its input from the disk or directly from primary memory, if it is still available. Variables containing processors’ outputs are automatically created and cleared internally as needed. The corresponding objects are referred to using node labels from the pipeline definition. This feature is very important during the development of a bioinformatic data analysis, where massive computations and several data files may be involved. The user is not forced to manually save and restore variables, thus preventing data inconsistency across development sessions. Moreover the definition of mnemonic names associated with derived resources is not necessary since the direct use of node names from the pipeline ensures a clear and simple way to identify them.

As mentioned, Pyleaf also supports parallel computing by exploiting multicore machines. For example, if the execution of the entire pipeline is requested (using the run method), Pyleaf will detect that the nodes visualize and analyze have no common ancestors and will run them in parallel.

Pyleaf also maintains a database that stores the source code of all the processors in order to ensure consistency between the current state of the pipeline and the produced resources. If a processor is modified, all dependant resources are automatically cleared (unless the user explicitly requests to *trust* an existing resource). Pyleaf also tracks files created by nodes marked with the flag “F” in order to verify that their content has not changed. This is currently performed by checking the time stamp of the most recent modification.

Finally, Pyleaf can automatically export the entire analysis as a hypertext document implementing our concept of bioinformatic protocol. The document includes a visualization of the pipeline, with nodes containing hypertext links to processor details. Such details include processor source code, documentation (automatically extracted from the original source code), execution time, and hypertext links to produced files together with their size. Overall time and space consumption as well as other statistics for the pipeline as a whole are also included. See Table 2 for an example.

**Table 2:** Example of statistics generated for the CNV analysis

	**Statistics for the entire analysis**
Number of nodes	24
Number of *F*-nodes	12
Total number of output files	76
Total size of output files	2.32G
Total CPU time required	03:02:15.25
**Statistics for a single node **(distMatGfx)	
Description	Produces an MDS visualization of the output of samplesDistMats
Output files	t_tani_distrib.pdf, t_tani_mds.pdf
Last build time	Sun Jan 8 03:51:56 2013
Required CPU time	00:01:25.08

The statistics are updated at each run or modification of any part of the pipeline. They are included in the final HTML protocol generated by Pyleaf. *F*-nodes are nodes whose output is written in one or more files on the disk. The total required time is estimated by summing up all the time required by single nodes, since they are usually ran across different sessions. Documentation for each node includes also source code, which is not reported in the Table. In the original document the file names are hyperlinked with actual files on the disk.

### Leaf and Python frameworks coexistence

The Leaf system is completely transparent to the user’s development environment. The LGL graph is defined as a multi-line Python string in the same source code implementing the Python functions that carry out each step of the analysis (see Figure 7). The processors in the pipeline are not implemented as structured objects, but rather as regular Python functions and the programmer does not need to use any special convention while writing his Python code. This framework allows the user to write plain and loosely structured Python code while still defining a high level scheme of the analysis. Both high and low levels of the analysis are managed together in the same source code. In fact, a project using Leaf typically includes a number of Python functions highlighted as nodes in the pipeline and others that are only implemented in the code, thus introducing a further mechanism of hierarchical structuring.

A common practice when extending an existing analysis within the Leaf environment is to exploit the protocol in order to easily set up the starting point for a new branch in the pipeline. Then the user requests to Pyleaf the resources that are necessary to the new branch. Pyleaf loads them from the disk or builds them on the fly running the necessary nodes from the pipeline and returns them to the user as regular Python objects. The user is able to define and test a new Python function and finally add it as a new node to the LGL graph in order to make it part of the pipeline. Conversely, existing pipeline nodes can be tested with new inputs by calling them as regular Python functions in a shell environment. In Leaf the usual direct call of Python functions seamlessly coexists with their indirect use as pipeline nodes.

## Application example

The Leaf system was developed to overcome practical problems that arose during a bioinformatic research project, the results of which are to be published soon. This application example included three main collaborating research units: application domain experts, data producers and data analysts (see Figure 1). As the research unit responsible for the data analysis, we were primarily concerned with: safely keeping primary resources provided by data producers as our ground-truth; easily identifying, storing and retrieving primary and derived resources in order to promptly respond to new requests from the other research groups; ensuring that all derived resources could be automatically reproduced starting from the primary ones; providing a clear report of all the steps of our analysis to be shared with other research groups; maintaining a documentation of our analysis making it easy to replicate, reuse in the future and back-trace in case of problems (this includes providing the execution provenance trace [8-10]). While our main goal was related to the development of computational methods, in this paper we describe practical issues concerning the development process of the analysis pipeline and how the Leaf system helped us overcome them efficiently.

**Figure 8:**
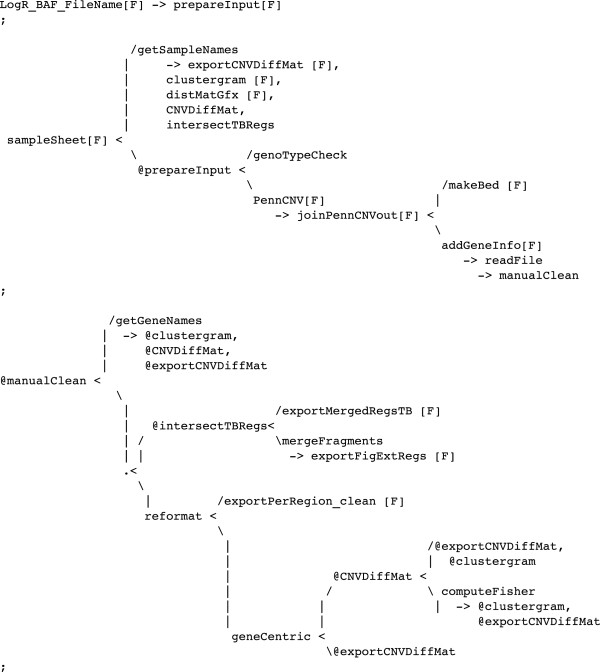
**LGL code for the CNV project.** The computational protocol in LGL for a real Copy Number Variation study. Compare with Figure 9. Refer to Figure 4 for basic syntax considering that the pipe (|) and newline characters are ignored by the compiler.

**Figure 9:**
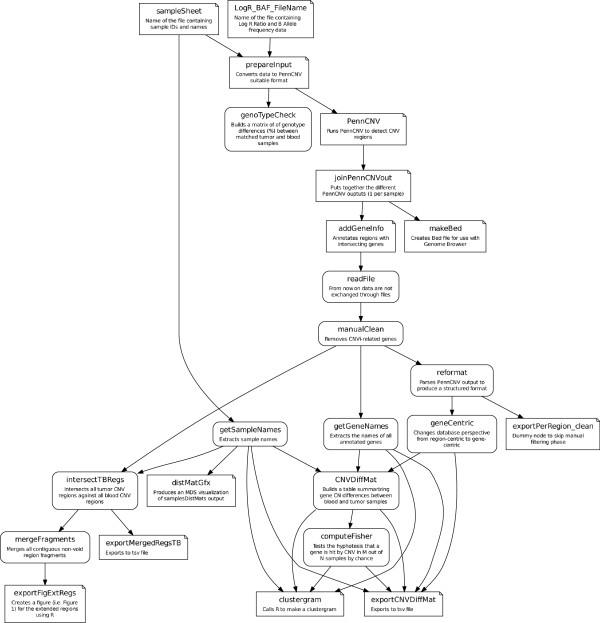
**Protocol’s graph for the CNV project.** Graphical representation of the LGL code of Figure 8. Leaf can internally use Graphviz [28] tools in order to automatically produce such representation starting from the output of the LGL compiler. Nodes with straight corners represent processors producing files.

The project involved a copy number variation (CNV [29]) analysis of a number of tissue samples. We used an existing software (PennCNV [30]) implemented as a Perl script for CNV detection. As an example of traceability enforcement, the primary resources were made compatible with the Perl script by an ad hoc converter routine written in Python. This conversion could have been more easily performed manually, but at the cost of breaking the *automaticity* rule (see “Concepts” section). The output of PennCNV suggested a number of hypotheses that were investigated through dedicated methods, which were heavily driven by partial results in a continuous feedback loop. The final computational pipeline for the analysis is shown in Figure 8 in the LGL language and in Figure 9 as the corresponding graphical visualization. Note that in the final pipeline the prepareInput processor calls a Unix Bash script, the PennCNV processor calls a Perl script, the clustergram processor calls an R script and all other processors call Python procedures. This is possible because of the high interoperability supported by the Python language, but is transparently included in the pipeline that provides a general overview of the analysis, evincing only the aspects that have been considered worth showing. The programmer used his preferred Python development framework to produce all the code for this study as well as the associated pipeline. In our case, even the Bash and R code were embedded in the Python source code, allowing Leaf to access and control all of the code implementing the pipeline nodes. R language is exploited through the dedicated RPy [31] Python library, while Bash scripts are encoded as Python multi-line strings and passed to system calls for execution.

The LGL code in the example (Figure 8) is quite involved and may seem difficult to work with. In our practice, complex LGL structures are created as the result of a code polishing phase, as soon as a portion of the pipeline has been assessed. Before this phase, a very simple syntax is adopted, with elementary structures incrementally appended to the graph. In fact, the entire LGL code in Figure 8 could be rewritten in LGL as a simple list of edges, as shown below:

A slightly more complex LGL statement defining the same structure could be:

where line breaks and indentation are discretionary. Since complex structures can be difficult to code, LGL provides simpler alternatives. The choice of syntax complexity level is left up to the programmer based on his skill level and preference.

The generated protocol for the latest version of the CNV project pipeline is available at the Leaf home page [12]. It is automatically generated by Pyleaf in HTML format. A sample of the statistics included in the protocol document is reported in Table 2.

## Conclusions

A balance between agility of code development and overall consistency and communicability in rapidly changing environments such as interdisciplinary research collaborations, is of fundamental importance, in regard to both methodology and efficiency. High-level tools are the most efficient when working on the general structure of the analysis, but make low-level interventions difficult. On the other hand, the use of formal design approaches can improve the robustness of a bioinformatic analysis, but at the cost of reducing responsiveness to change.

The Leaf system, like other tools in the same category, allows for low-level access to implementation details, but still provides tools for the management of a light-weight, loosely structured data flow layer. In particular Leaf supports a dedicated pipeline definition language, LGL, whose flexible coding style allows the programmer to choose his preferred balance between easy to read and easy to write code.

To our knowledge, Leaf is the only pipeline manager that allows the definition of both the pipeline and its nodes in the same source file, while at the same time keeping them separated. Besides the general properties connected with the design of the Leaf system, we also highlighted some features supported by the Python implementation of Leaf that are not present in similar tools, such as the monitoring of source code, which allows for consistency checks between code and resources. Additional features already present in other tools have been further developed in Leaf, such as the generation of pipeline documentation in the form of a hypertext, including links to the files produced during pipeline execution and statistics about time and space requirements detailed for each node, to name a few.

In our opinion, both the design philosophy and the implemented features of Leaf make it a valuable alternative to other pipeline management systems.

## Availability and requirements

**Project name:** Leaf

**Project home page:**http://www.neuronelab.dmi.unisa.it/leaf

**Operating system(s):** Linux, Windows. Mac OS under development.

**Programming language(s):** C++, Python.

**Other requirements:** Python ≥ 2.6.

**License:** MIT.

**Any restrictions to use by non-academics:** None.

## Abbreviations

LGL: Leaf graph language; DAG: Directed acyclic graph.

## Competing interests

The authors declare that they have no competing interests.

## Authors’ contributions

FN designed and implemented the Leaf system. RT provided design and implementation feedback. RMC provided application domain feedback. RT and RMC supervised the project. All authors contributed in writing the manuscript and read and approved the final version.
